# Spatiotemporal Characteristics and Health Risk Assessment of Heavy Metals in PM_2.5_ in Zhejiang Province

**DOI:** 10.3390/ijerph15040583

**Published:** 2018-03-24

**Authors:** Xiaofeng Wang, Shengliang He, Shuchang Chen, Yongli Zhang, Aihong Wang, Jinbin Luo, Xialiang Ye, Zhe Mo, Lizhi Wu, Peiwei Xu, Gaofeng Cai, Zhijian Chen, Xiaoming Lou

**Affiliations:** 1 Zhejiang Provincial Center for Disease Prevention and Control, Binsheng Road 3399, Hangzhou 310051, China; xfwang@cdc.zj.cn (X.W.); shlhe@cdc.zj.cn (S.H.); zhmo@cdc.zj.cn (Z.M.); lzhwu@cdc.zj.cn (L.W.); pwxu@cdc.zj.cn (P.X.); 2 Hangzhou Center for Disease Control and Prevention, Mingshi Road 568, Hangzhou 310021, China; cschzcdc@163.com; 3 Zhoushan Center for Disease Control and Prevention, Wengshan Road 568, Zhoushan 316021, China; zhangcdc@139.com; 4 Ningbo Center for Disease Control and Prevention, Yongfeng Road 237, Ningbo 315010, China; wangah@nbcdc.org.cn; 5 Jinhua Center for Disease Control and Prevention, Jinou Road 1366, Jinhua 321002, China; jhcdcljb@163.com; 6 Lishui Center for Disease Control and Prevention, Shouerfu Road 28, Lishui 323000, China; zjsyyxl@126.com

**Keywords:** PM_2.5_, heavy metals, health risk assessment, pollution assessment

## Abstract

The spatiotemporal characteristics and human health risks of 12 heavy metals (Al, As, Be, Cd, Cr, Hg, Mn, Ni, Pb, Sb, Se, and Tl) in fine particulate matter (PM_2.5_) in Zhejiang Province were investigated. The annual average PM_2.5_ concentration was 58.83 µg/m^3^ in 2015 in Zhejiang. Element contents in PM_2.5_ varied greatly with the season and locations. Al, Pb, and Mn were the most abundant elements among the studied metal(loid)s in PM_2.5_. The non-carcinogenic risks of the 12 elements through inhalation and dermal contact exposure were lower than the safe level for children and adults. However, there were potential non-carcinogenic risks of Tl, As, and Sb for children and Tl for adults through ingestion exposure. The carcinogenic risks from As, Be, Cd, Cr, Pb, and Ni through inhalation exposure were less than the acceptable level (1 × 10^−4^) for children and adults. Pb may carry a potential carcinogenic risk for both children and adults through ingestion. More attention should be paid to alleviate non-carcinogenic and carcinogenic health risks posed by particle-bound toxic elements through ingestion exposure.

## 1. Introduction

Atmospheric particulate matter, as the key component of the hazy episode, has become one of the top environmental issues in China. Particulate matter in air is one of the serious air pollutants worldwide and has been classified as a group 1 carcinogenic agent by the International Agency for Research on Cancer (IARC) [1]. However, the threshold of particulate matter (PM) levels on adverse health effects has been identified [2].

China has been suffering from severe haze-pollution in recent years, especially in the fastest growing economic development regions such as the Yangtze River Delta (YRD) region [3,4]. As one of the main provinces in YRD, the duration and impact of hazy days in Zhejiang Province kept increasing from the 1970s, especially from 2000 [5,6]. For example, the extremely severe haze in December 2013 lasted for a long time and covered the whole province [7,8]. In 2013, the annual average concentrations of PM_2.5_ were 93 µg/m^3^, 92 µg/m^3^, 84 µg/m^3^, 76 µg/m^3^, 72 µg/m^3^, and 61 µg/m^3^ in Jinhua, Hangzhou, Ningbo, Quzhou, Wenzhou, and Lishui of Zhejiang Province, respectively [6]. A recent study in Delhi of India demonstrated PM_2.5_ levels between 54 and 277 µg/m^3^ [9]. However, the annual average of the PM_2.5_ concentration recorded in those places was generally quite low, about 13 µg/m^3^ in California and 15 µg/m^3^ in New York [10,11]. Another study focused on European countries reported a level of 0.030 mg/m^3^ at the Italian site called Ispra [12], while the daily mean level of PM_2.5_ was 7.79 µg/m^3^ in Adelaide, South Australia for the period 2003–2013 [13]. Besides, the mean level of PM_2.5_ was 11 µg/m^3^ in Toronto, Canada [14]. The average levels of PM_2.5_ in China were generally higher than those in developed countries. Therefore, it is crucial to evaluate the spatiotemporal characteristics and health risk assessment of PM_2.5_, especially of heavy metals in Zhejiang.

According to categories defined by the Environmental Protection Agency (EPA), particulate matter is mainly divided into (i) coarse particulate matter (PM_10_) with an aerodynamic diameter of 10 μm and (ii) fine particulate matter (PM_2.5_) with an aerodynamic diameter of 2.5 μm [15]. Compared to PM_10_, PM_2.5_ can be inhaled and travel deeply into the respiratory tract and the alveoli and, eventually, is deposited in the respiratory system or further drawn into capillaries and transferred to other organs or systems through circulation [16]. Therefore, elevated levels of PM_2.5_ in the atmosphere have become a top environmental concern and may cause a wide range of health problems [17]. PM_2.5_ primarily comes from a wide range of sources with different toxicities, including traffic emissions, biomass combustion, industrial and agricultural activities, and crustal origination [16,18,19]. It also has a large surface area and acts as a carrier of crustal elements and trace elements, including heavy metals (e.g., Cr, V, Ni, Cu, Zn, Sb, and Pb), organic and elemental carbon (OC and EC), and microorganisms (bacteria and viruses). Among them, heavy metals from atmospheric particulate matter may accumulate in human bodies and pose a potential threat to public health due to lack of bioavailability, biodegradability, and persistence, especially for children [20,21,22,23,24]. The chemical fractionation and health risks of heavy metals in PM_2.5_ can offer more valuable information than the analysis of total concentrations to the policymaker.

In the present study, PM_2.5_ was collected from five types of development cities in Zhejiang Province monthly. The main objectives of this study were as follows: (1) to investigate the spatiotemporal distributions of heavy metals in PM_2.5_ from different cities in Zhejiang Province; and (2) to evaluate the carcinogenic and non-carcinogenic health risks associated with heavy metals through inhalation, ingestion, and dermal contact exposure.

## 2. Materials and Methods

### 2.1. Investigation Fields

**Figure 1:**
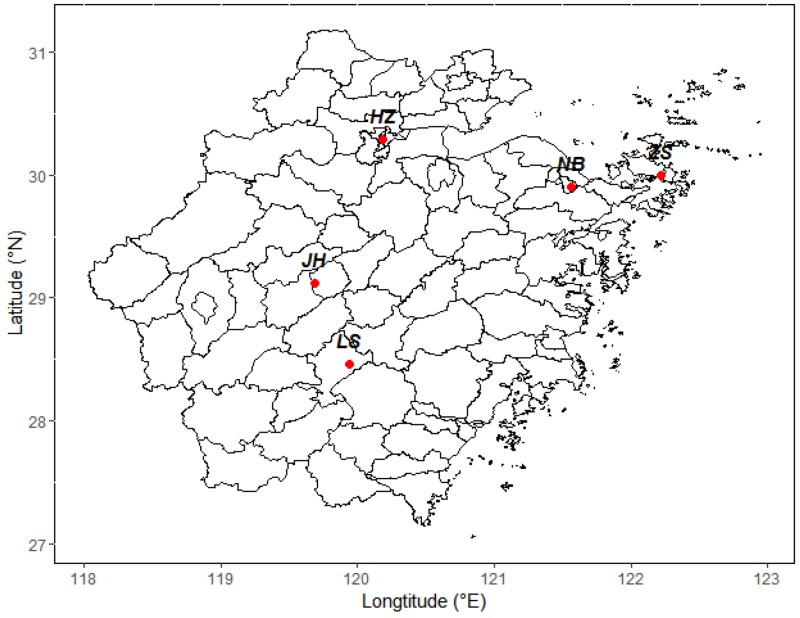
Location of the survey areas in Zhejiang Province, China.

In this study, five cities in Zhejiang province were included: Hangzhou, Ningbo, Zhoushan, Jinhua, and Lishui (Figure 1). Hangzhou (HZ) is the capital of Zhejiang Province and is one of the prosperous cities in the Yangtze Delta. Hangzhou is a famous tourist city with a population of 9.02 million in 2015, and there are more than 2.6 million cars. Ningbo (NB) is the world’s fourth largest port city, located on the eastern coast of Zhejiang Province. Ningbo is an important chemical industry base in the Yangtze Delta and is famous for its textile and garment industries. Zhoushan (ZS) is an island city located in the northeast of Zhejiang Province and has the largest seafood-production, processing, and marketing base in China. Jinhua (JH) is located in the middle of Zhejiang Province and is hill-and-basin in landform. The pillar industry in Jinhua is the manufacturing of metal products and medicine. Lishui (LS) is located in the southwest of Zhejiang Province, mainly in mountainous and hilly terrain. Lishui is called a forest city, with forest coverage of 80.79%.

### 2.2. Sample Collection

The sampling site was set on the rooftop of a building in each city, which was 12–15 m above ground. PM_2.5_ samples were collected on 47-mm quartz media filters (QZ47DMCAN, MTL, Minneapolis, MN, USA) using a mid-volume sampler (MVS6, LECKEL, Berlin, Germany) at a flow rate of 38.3 L/min. Sampling was conducted from the 10th to the 16th of every month, and the collection duration for each sample was 23 h (starting at 9:00 a.m. local time each day and ending at 8:00 a.m. next day). Two samplers were used in each sampling site, one for the experiment group and the other for a blank control group. The blank samples were only collected on the loaded filters without operation on the 10th and 16th every month. A total of 540 samples was collected from January to December 2015. The filters were weighed by an automated filter weighing system (WZZ-02, Weizhizhao, Hangzhou, China) before and after sampling to determine the mass of PM_2.5_. The filters were subsequently sealed in a filter holder and stored at −20 °C until analysis. Meteorological parameters such as wind speed, wind direction, temperature, and humidity were also recorded at the time of sample collection. 

### 2.3. Sample Analysis

Heavy metals and metalloids (Al, As, Be, Cd, Cr, Hg, Mn, Ni, Pb, Sb, Se, and Tl) were determined using inductively coupled plasma mass spectrometry (ICP-MS, Thermo Fisher Scientific, ICAP Qc ICP-MS). The filter was placed in the bottom of a 15-mL centrifuge tube with 10 mL of 5% nitric acid. The tube was incubated at 70 °C, with ultrasonic extraction, for 3 h. After cooling, the samples were centrifuged at 4500 rpm/min for 5 min. The separated supernatant liquid in the top layer was filtrated through a 0.45-μm membrane and subjected to analyses of heavy metals by ICP-MS. QA/QC included reagent blanks; analytical duplicates; and analysis of the standard reference material (Scandium (GSB04-1750-2004), Germanium (GSB04-1728-2004), Indium (GSB04-1731-2004), and Yttrium (GSB04-1788-2004), which were from the National Center of Analysis and Testing for Nonferrous Metals and Electronic Materials, China. The concentration of each standard element was 10 μg/L in 2% HNO_3_. The concentration of standard curve of each element was 2–100 μg/L (the concentration of Al was 2–1000 μg/L). The element recovery percentage from the standard reference material was between 82% and 108%.

### 2.4. Exposure Assessment

The three major pathways of residential exposure to heavy metals in PM_2.5_ are ingestion, inhalation, and dermal contact [16,25]. The exposure dose through each of the three pathways was calculated according to the Human Health Evaluation Manual (Part A), Supplemental Guidance for Dermal Risk Assessment (Part E), and Supplemental Guidance for Inhalation Risk Assessment (Part F) [26,27,28]. Exposure dose was expressed in terms of a daily intake and calculated separately for each element and pathway. The equations were as follows:

CDI = (C × IR × EF × ED × CF)/(BW × AT)(1)

EC = (C × ET × EF × ED)/ATn(2)

DAD = (C × SA × AF × ABS × EF × ED × CF)/(BW × AT)(3)

in which CDI = chemical daily intake through ingestion (mg/kg), EC = exposure concentration through inhalation (µg/m^3^), and DAD = dermally absorbed dose (mg/kg). The descriptions and values of all the parameters are given in Table 1.

### 2.5. Risk Characterization

Risk characterization was evaluated for non-carcinogenic and carcinogenic risks. The non-carcinogenic risk of a single contaminant in an exposure pathway was evaluated by the hazard quotient (HQ). The hazard index (HI), the sum of HQ, was used to assess the overall potential for non-carcinogenic effects posed by more than one chemical. An HI < 1 indicates that the risk of non-carcinogenic effects are not significant and sometimes may be neglected. Conversely, an HI ≥ 1 indicates that non-carcinogenic effects are possible, with a probability that tends to increase as the value of HI increases [34]. Carcinogenic risks (CRs) is the probability of an individual developing any type of cancer from lifetime exposure to carcinogenic hazards. The acceptable or tolerable risk is from 1 × 10^−6^ to 1 × 10^−4^ [34].

HQ and CR posed by heavy metals in PM_2.5_ by ingestion, dermal contact, and inhalation were calculated by the following equations:

HQ = CDI/RfDo = DAD/(RfDo × GIABS) = EC/(RfCi × 1000)(5)

CR = CDI × SFo = DAD × (SFo/GIABS) = IUR × EC(6)

in which RfDo is oral reference dose (mg/kg/day), RfCi is inhalation reference concentration (mg/m^3^), GIABS is gastrointestinal absorption factor, SFo is oral slope factor ((mg/kg/day)^−1^), and IUR is inhalation unit risk ((µg/m^3^)^−1^).

According to the classification defined by the IARC, As, Be, Cd, Cr(VI), and Ni are group I carcinogenic agents; Sb, Pb, and Hg are group 2B; and Se is group 3 [1]. Al, Mn, and Tl are not found in the classified group orders [1]; therefore, their carcinogenic risks in PM_2.5_ were not investigated in the present study. The SFo, RfDo, RfC, GIABS, and IUR values for As (inorganic), Be, Cd (diet), Cr (VI), Ni (refinery dust), and Pb (acetate) were found in the regional screening level (RSL) summary tables provided by the U.S. EPA [35].

## 3. Results and Discussion

### 3.1. Spatiotemporal Distributions of PM_2.5_ and Heavy Metals

**Figure 2:**
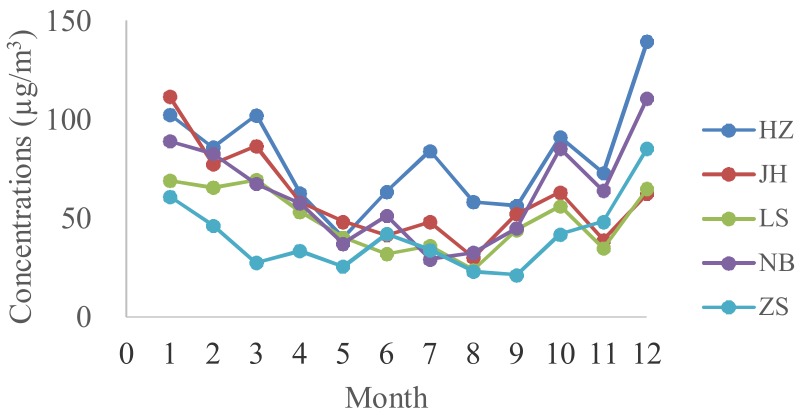
Monthly average concentrations of PM_2.5_ in the five cities.

The monthly average concentrations of PM_2.5_ in the 5 cities are shown in Figure 2. The ranges of monthly average concentrations of PM_2.5_ are 40.25–139.67 µg/m^3^, 30.16–111.63 µg/m^3^, 29.29–110.75 µg/m^3^, 23.88–69.50 µg/m^3^, and 21.38–85.33 µg/m^3^ in HZ, JH, NB, LS, and ZS, respectively. The annual average PM_2.5_ concentrations (58.83 µg/m^3^) in Zhejiang were higher than the Ambient Air Quality standard of China (GB 3095–2012) (35 µg/m^3^, level II). However, the concentration is lower than that in Chengdu (165.1 µg/m^3^), Zhengzhou (175 µg/m^3^), and Agra (104.9 µg/m^3^) [36,37,38], and close to that in Shanghai (62.25 µg/m^3^) and Chengdu (63 µg/m^3^) [39,40].

**Figure 3:**
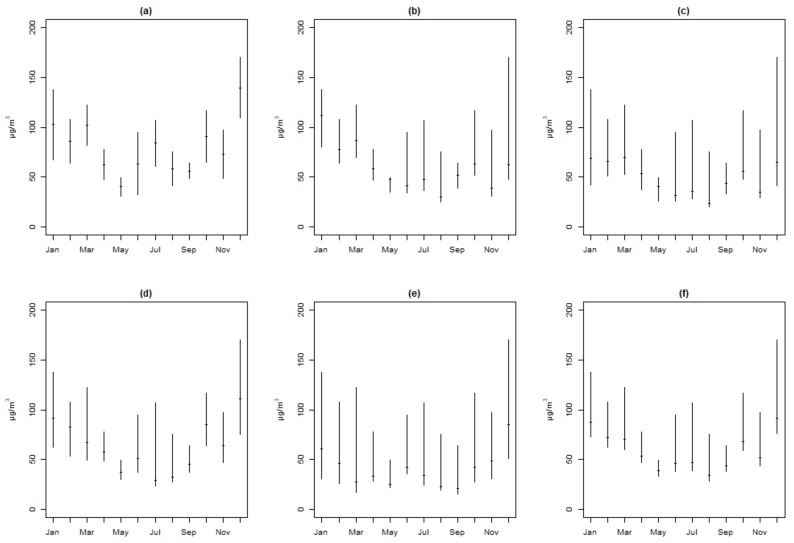
Seasonal variation of PM_2.5_ concentrations in HZ (**a**), JH (**b**), LS (**c**), NB (**d**), ZS (**e**), and Total (**f**).

In general, the PM_2.5_ concentrations in HZ were higher than those in other cities, and ZS showed the lowest concentrations. Figure 3 provided evidence of a clear seasonal variation. The seasonal variation of PM_2.5_ concentrations showed very similar patterns in all five cities (Figure 3a–e). Median values of PM_2.5_ concentrations are higher in cold months and lower in hot months, as can be seen for the PM_2.5_ concentration (all cites in Figure 3f). The results were similar to the data reported by Sun et al. [41]. Deng et al. reported that good air quality usually occurred in the period from May to October in HZ [8]. Previous study by Xiao et al. demonstrated that the haze days were more frequent in spring (from March to May) and winter (from December to February) and less frequent in summer (from June to August) and autumn (from September to November) [5]. The reason might be ascribed to poor conditions for the dispersion of air pollution and the influence of fossil fuel combustion for power and heating in the north in the cold months [42]. During winter months, the surface wind speed is typically low, allowing for greater stratification, which favors the accumulation of air pollution [43].

**Figure 4:**
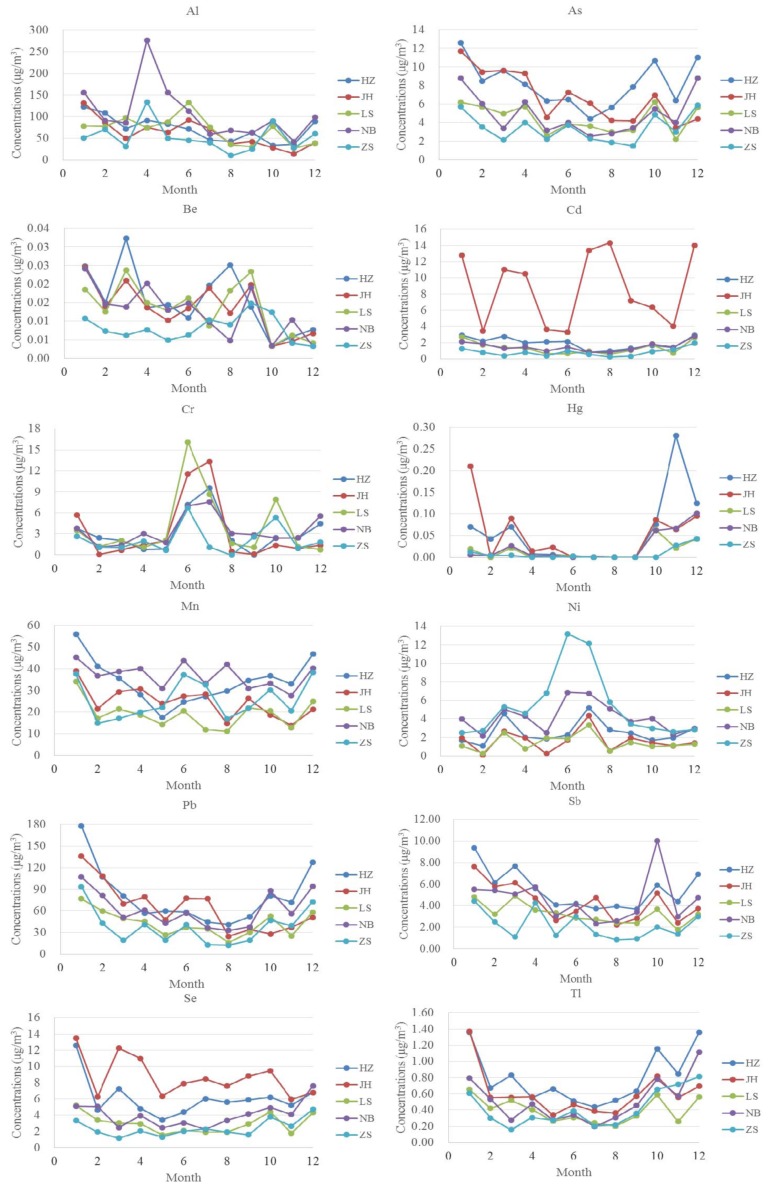
Monthly variations of the 12 element concentrations in PM_2.5_.

The descriptive statistics of the 12 studied elemental contents in PM_2.5_ are listed in Table 2. In general, the contents of Al, Pb, and Mn were generally higher than those of the other elements in PM_2.5_, which were consistent with previous studies [17,25,44]. The metal concentrations in PM_2.5_ varied over sampling sites. As, Pb, Sb, and Tl are dominant in HZ, whereas JH has higher concentrations of Cd and Se. Al, Cr, and Ni are relatively higher in NB, LS, and ZS, respectively. Monthly variations of the 12 element concentrations in PM_2.5_ are summarized in Figure 4. Although the metal concentrations in PM_2.5_ varied over the sampling time, the monthly changes in trend of metal concentrations was similar to those of PM_2.5_ concentrations. Most of the metal concentrations showed a similar change trend in different sampling sites except for Cd. The Cd concentration in JH was several times higher than that in other cities and varied irregularly over the sampling time.

### 3.2. Non-Carcinogenic Risk Assessment of Toxic Elements in PM_2.5_

Table 3 shows the non-carcinogenic risks from toxic elements in PM_2.5_ through ingestion, inhalation, and dermal contact exposure in the five cities. The HQ values for Al, As, Be, Cd, Co, Cr, Hg, Mn, Ni, Sb, Se, and Tl through inhalation and dermal contact exposure for both children and adults were all lower than the safe level (=1), indicating no significant non-carcinogenic risks from the inhalation and dermal contact exposure for each single element. In addition, the HI values through inhalation and dermal contact exposure were all lower than the safe level for children and adults. These findings indicated that the integrated effects of multi-elemental exposure to PM_2.5_ through inhalation and dermal contact exposure may not result in non-carcinogenic adverse effects to residents.

HQ values of Tl (10.31), As (3.56), Sb (1.83) for children, and Tl (1.44) for adults through ingestion exposure were higher than the safe level, which were obviously higher than those for other elements. HI values through ingestion exposure were all higher than the safe level for children (16.40) and adults (2.30), which mainly resulted from Tl, As, and Sb. The results indicated the potential non-carcinogenic risks to local residents through ingestion exposure.

The HQ values of heavy metals in PM_2.5_ for children and adults due to different exposure pathways decreased in the following order: ingestion > inhalation > dermal contact. The contribution of HI_Ing_ to the HI was the highest (94.39%) for children, whereas the contribution of HI_Der_ to the HI could be neglected (only 0.026%). The contributions of HI_Ing_, HI_Inh_, and HI_Der_ to the HI were 69.89%, 29.52%, and 0.59% for adults, respectively. These results indicated that ingestion was the primary pathway for heavy metals in PM_2.5_ and might pose higher non-carcinogenic risks to both children and adults. This result is consistent with other investigations [45]. The HQ values of ingestion, inhalation, and dermal contact exposure for the studied toxic elements decreased in the following order: Tl > As > Sb > Cd > Se > Cr > Mn > Ni > Al > Hg > Be, Mn > As > Cd > Ni > Sb > Al > Cr > Be > Se > Hg, and Sb > As > Tl > Cr > Cd > Ni > Se > Be > Mn > Al > Hg, respectively. Compared to HI for adults (3.28), the HI values for children (17.38) were significantly higher, suggesting that children may suffer more harmful health risks from heavy metals in PM_2.5_. Obviously, the higher ingestion rate may account for the higher non-carcinogenic risks to children.

**Figure 5:**
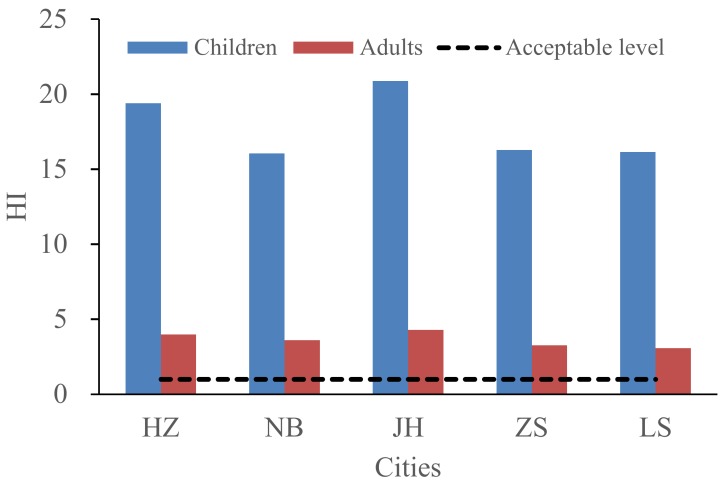
HI values for children and adults in the five cities.

**Figure 6:**
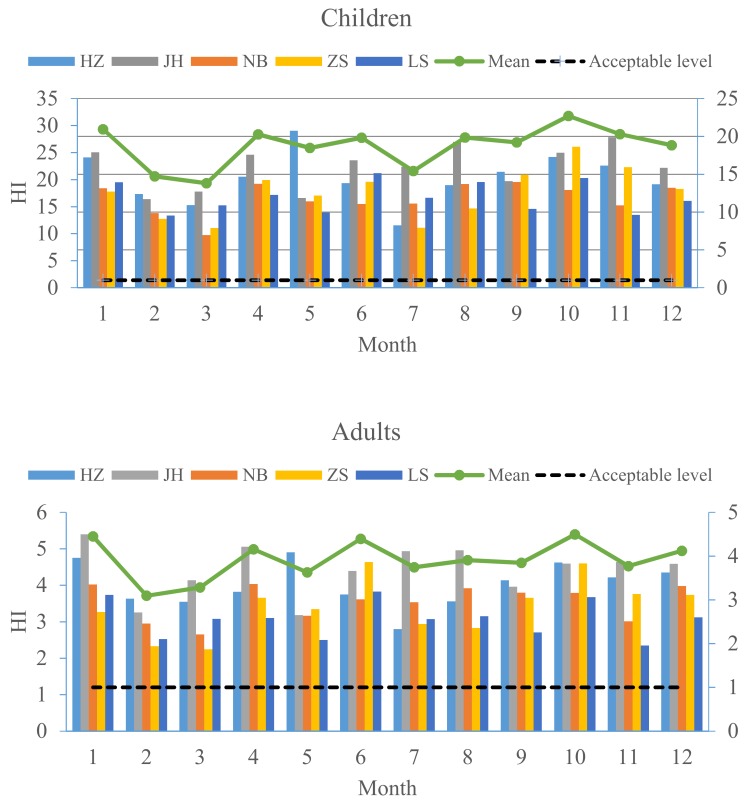
Monthly variations of HI values for children and adults in the five cities.

In this study, the HI values for non-carcinogenic risk varied among different cities. Residents living in JH (20.88) and HZ (19.39) faced a higher health risk than residents living in NB (16.05), ZS (16.28), or LS (16.14) (Figure 5). The HI values of the three exposure pathways had the same trends: ingestion > inhalation > dermal contact in all cities. In addition to the ingestion exposure, the elements played different roles in the HI in each city. The HQ values of the top three heavy metals for exposure through inhalation, ranked in deceasing order, were Mn > As > Cd in HZ, Mn > Ni > As in NB, Cd > Mn > As in JH, Mn > Ni > As in ZS, and Mn > As > Cd in LS. However, the HQ values through dermal exposure ranked Sb > As > Tl in HZ, NB, JH, and LS, and Tl > As > Sb in ZS. Figure 6 shows the monthly variations of HI values for children and adults in the five cities. HI values in February, March, May, and July were relatively lower than those in other months.

### 3.3. Carcinogenic Risk Assessment of Toxic Elements in PM_2.5_

The carcinogenic risks from As, Be, Cd, Cr, Pb, and Ni through inhalation exposure were less than 1 × 10^−4^ for children and adults, and the integrated risks of those elements were also within the acceptable level (1 × 10^−4^). The results indicated that the carcinogenic risk posed by these toxic elements to local residents through inhalation was acceptable. Because of the lack of carcinogenic slope factors for Be, Cd, and Ni, the carcinogenic risks for these three elements through ingestion and dermal contact were not estimated. For ingestion, the CR values of Cr were under the acceptable level for both children and adults. The value of As through ingestion was slightly higher than the acceptable level for children (1.37 × 10^−4^) but lower than the acceptable level for adults (7.70 × 10^−5^). Pb may have potential carcinogenic risk for both children (2.57 × 10^−4^) and adults (1.44 × 10^−4^). The carcinogenic risks from As, Cr, and Pb through dermal contact exposure were less than 1 × 10^−4^ for children and adults, and the integrated risks of those elements were also within the acceptable level (1 × 10^−4^).

The carcinogenic risks posed by the toxic elements in PM_2.5_ were 4.64 × 10^−4^ for children and 3.12 × 10^−4^ for adults (Table 4) on average. Similar to HI values, CR values for children were also higher compared to adults. The highest CR value for children occurred in JH (5.11 × 10^−4^), followed by HZ (5.08 × 10^−4^), NB (4.87 × 10^−4^), LS (4.63 × 10^−4^), and ZS (4.01 × 10^−4^). The CR values for adults decreased in the following order: NB (3.57 × 10^−4^), JH (3.44 × 10^−4^), HZ (3.44 × 10^−4^), LS (3.28 × 10^−4^), and ZS (2.67 × 10^−4^). These values were higher than the acceptable level (1 × 10^−4^), implying that the carcinogenic risk to the residents posed by the toxic elements in PM_2.5_ is not negligible in the five cities.

The CR values for the different exposure pathways of heavy metals in PM_2.5_ for children and adults decreased in the following order: ingestion > dermal contact > inhalation. The contribution of CR_Ing_ to the CR was the highest (86.93%) for children, followed by CR_Der_ (11.45%) and CR_Inh_ (1.62%). The contribution of CR_Ing_, CR_Der_, and CR_Inh_ to the CR were 72.26%, 18.12%, and 9.63% for adults, respectively. The results indicated that ingestion was the primary pathway for heavy metals in PM_2.5_ and posed a higher carcinogenic risk to both children and adults.

**Figure 7:**
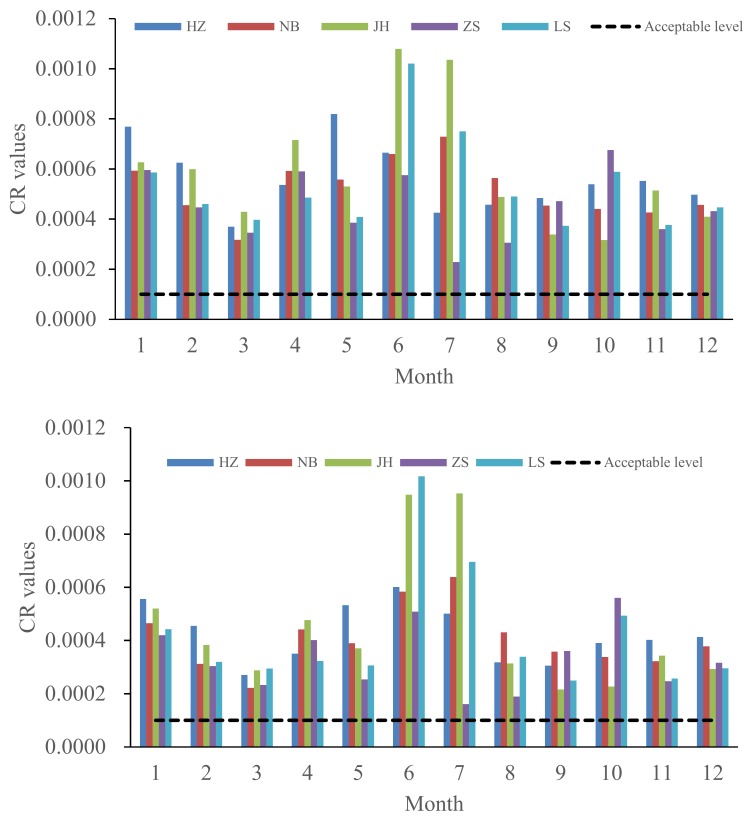
Monthly variations of CR values for children and adults in the five cities.

The distribution of CR values by month varied in each city, with higher values in January, May, and June in HZ; January, April, May, June, July, and August in NB; June and July in both JH and LS; and January, April, June, and October in ZS (Figure 7). The higher CR values were almost attributed to the higher concentrations of Pb in each city.

### 3.4. Uncertainty and Limitation of Health Risk Assessment

The risk assessment models developed by the U.S. EPA were adopted in this study for the estimation of cancer and non-cancer risks of heavy metals in PM_2.5_. Although the heavy-metal concentrations in PM_2.5_ were detected 7 days monthly in this study, uncertainties could be caused by the day-to-day variations in concentrations and composition of heavy metals in PM_2.5_ for meteorological factors such as the wind speed, wind direction, and rainfall.

The spatial variation of heavy metals in PM_2.5_ is influenced by the pollution source. In the present study, only one sampling site (the traditional center district) was set up in each city. If more sampling sites were built in the different areas, such as industrial, commercial, residential, and scenic areas, the results of health risks for the local residents may be more representative and accurate.

Uncertainties could also exist in the risk assessment of the exposure method. These include a lack of values assigned to population exposure variables. For example, the exposure time and exposure frequency showed significant regional or individual differences. The concentrations and composition of heavy metals in PM_2.5_ are very different between outdoors and indoors. In general, the concentrations of heavy metals in PM_2.5_ in the room are lower than those outside, which may overestimate the health risks.

In addition, the RfD, IUR, and SF of a chemical are used in this study, which may also result in the uncertainty of health risks. For example, Cr toxicity is directly dependent on its valence state. Cr (III) is generally regarded as an essential trace element in the human body and is the basis of glucose tolerance factor [46]. Conversely, Cr (VI) has been determined to be a human carcinogen. The RfD, IUR, and SF of Cr (VI) was used in this study, which may overestimate the actual hazards they pose to humans. The estimated number of people who may develop cancer in this study is also highly uncertain because of individual differences. Although it contains such uncertainties and limitations, the study still provides a valuable evaluation of the health risks associated with the exposure to heavy metals in PM_2.5_.

## 4. Conclusions

The spatiotemporal characteristics and health risk assessment of heavy metals and metalloids (Al, As, Be, Cd, Cr, Hg, Mn, Ni, Pb, Sb, Se, and Tl) in PM_2.5_ in Zhejiang Province were investigated in this study. The average PM_2.5_ concentrations appeared to have significant seasonality. Comparatively higher concentrations of PM_2.5_ occurred in cold months (January and December), whereas the lowest concentrations were recorded in hot months (June, July, and August) in all five cities. Al, Pb, and Mn were the most abundant elements among the studied metals in PM_2.5_. The integrated effects of multi-elemental exposure to PM_2.5_ through inhalation and dermal contact exposure were all lower than the safe level (=1). However, there were potential non-carcinogenic risks to local residents through ingestion exposure. Among the three exposure pathways, ingestion was the primary pathway for heavy metals in PM_2.5_ and might pose higher non-carcinogenic risks to both children and adults. Pb may pose a potential carcinogenic risk for both children (2.57 × 10^−4^) and adults. Comparatively, children may suffer more harmful health risks from heavy metals in PM_2.5_. Therefore, the carcinogenic and non-carcinogenic risks to children and adults from the toxic elements in PM_2.5_ must be considered. The concentrations of heavy metals and health risks provided more information to the policymaker for atmospheric pollution control. We hope these results will be useful both for residents to take protective measures and help raise focus on enforcing more stringent limitations on air pollution.

## Figures and Tables

**Table 1:** Input parameters and abbreviations for cancer and non-cancer exposure assessment.

Parameter	Notation	Unit	Value		References
			Children	Adults	
Metal concentrations in PM_2.5_	C	µg/m^3^	Table 2		This study
Average lifetime	ATn	hours	ED × 365 × 24 (for non-carcinogens) 70 × 365 × 24 (for carcinogens)	ED × 365 × 24 (for non-carcinogens) 70 × 365 × 24 (for carcinogens)	[29]
Averaging lifetime	AT	days	ED × 365 (for non-carcinogens) 70 × 365 (for carcinogens)	ED × 365 (for non-carcinogens) 70 × 365 (for carcinogens)	[29]
Body weight	BW	kg	15.9	56.8	[30]
Conversion factor	CF	mg/kg	10^−6^	10^−6^	[29]
Exposure duration	ED	year	6	24	[29]
Exposure frequency	EF	days/year	350	350	[31]
Exposure time	ET	h/day	24	24	[29]
Ingestion rate	IR	mg/day	200	100	[29]
Skin surface area adherence that contacts the airborne particulates	SA	cm^2^	1600	4350	[31]
Skin adherence factor for the airborne particulates	AF	mg/cm^2^	0.2	0.07	[32]
Dermal absorption factor	ABS	/	0.03 (As), 0.1 (Pb), 0.001 (Cd), 0.01 (other metals)		[33]

**Table 2:** Concentrations of PM_2.5_ (µg/m^3^) and 12 toxic heavy metals in PM_2.5_ (ng/m^3^) from 5 cities.

City	PM_2.5_	Al	As	Be	Cd	Cr	Hg	Mn	Ni	Pb	Sb	Se	Tl
HZ	78.73 ± 39.85	71.16 ± 45.47	8.08 ± 4.19	0.02 ± 0.01	1.91 ± 1.42	3.14 ± 3.05	0.06 ± 0.12	34.01 ± 18.65	2.55 ± 1.91	78.79 ± 53.77	5.44 ± 3.11	6.06 ± 3.47	0.78 ± 0.47
JH	61.72 ± 32.43	63.52 ± 44.96	7.02 ± 5.45	001 ± 0.01	8.81 ± 11.98	3.57 ± 5.13	0.05 ± 0.08	25.37 ± 12.38	1.70 ± 1.86	66.48 ± 50.50	4.45 ± 2.33	8.96 ± 4.53	0.62 ± 0.40
NB	62.89 ± 35.86	108.15 ± 85.92	4.88 ± 3.30	0.01 ± 0.01	1.50 ± 0.91	3.42 ± 3.52	0.02 ± 0.04	36.80 ± 15.18	4.09 ± 2.78	61.88 ± 38.46	4.61 ± 3.90	4.06 ± 2.92	0.51 ± 0.37
ZS	41.88 ± 31.62	52.88 ± 44.90	3.48 ± 2.76	0.01 ± 0.01	0.83 ± 0.85	2.14 ± 2.25	0.01 ± 0.02	26.29 ± 22.03	5.30 ± 4.39	40.28 ± 49.54	2.25 ± 2.59	2.44 ± 1.93	0.42 ± 0.35
LS	48.99 ± 24.44	69.32 ± 56.64	4.39 ± 2.58	0.01 ± 0.01	1.34 ± 1.01	3.95 ± 6.08	0.01 ± 0.02	18.99 ± 10.32	1.44 ± 1.25	42.33 ± 25.08	3.22 ± 1.76	2.93 ± 1.65	0.39 ± 0.24
Mean	58.83 ± 35.36	72.61 ± 60.05	5.60 ± 4.20	0.01 ± 0.01	3.02 ± 6.45	3.25 ± 4.29	0.03 ± 0.07	28.18 ± 17.33	2.99 ± 3.03	58.05 ± 47.03	4.00 ± 3.02	4.98 ± 3.95	0.55 ± 0.40

**Table 3:** Non-carcinogenic risks from toxic elements in PM_2.5_.

Toxic Elements	95%UCL		CDI (mg/kg-Day)		EC (μg/m^3^)		DAD (mg/kg)		Non-Carcinogenic Risks (HQ)							
	(ng/mg)	(ng/m^3^)	Child	Adult	Child	Adult	Child	Adult	Child-Ing	Adult-Ing	Child-Inh	Adult-Inh	Child-Der	Adult-Der	Child	Adult
Al	1.59 × 10^3^	7.80 × 10^1^	1.18 × 10^−2^	1.66 × 10^−3^	4.54 × 10^−2^	4.54 × 10-^2^	1.90 × 10^−4^	5.05 × 10^−5^	1.18 × 10^−2^	1.66 × 10^−3^	9.09 × 10^−3^	9.09 × 10^−3^	1.39 × 10^−6^	5.94 × 10^−6^	2.09 × 10^−2^	1.08 × 10^−2^
As	1.07 × 10^2^	5.97 × 10^0^	1.07 × 10^−3^	1.50 × 10^−4^	4.24 × 10^−3^	4.24 × 10^−3^	5.13 × 10^−5^	1.37 × 10^−5^	3.56 × 10^0^	4.99 × 10^−1^	2.12 × 10^−1^	2.12 × 10^−1^	1.26 × 10^−3^	5.36 × 10^−3^	3.78 × 10^0^	7.16 × 10^−1^
Be	2.78 × 10^−1^	1.37 × 10^−2^	2.16 × 10^−6^	3.02 × 10^−7^	8.55 × 10^−6^	8.55 × 10^−6^	3.46 × 10^−8^	9.21 × 10^−9^	1.08 × 10^−3^	1.51 × 10^−4^	4.27 × 10^−4^	4.27 × 10^−4^	1.81 × 10^−5^	7.73 × 10^−5^	1.53 × 10^−3^	6.56 × 10^−4^
Cd	6.31 × 10^1^	3.59 × 10^0^	3.58 × 10^−4^	5.01 × 10^−5^	1.42 × 10^−3^	1.42 × 10^−3^	5.73 × 10^−7^	1.53 × 10^−7^	3.58 × 10^−1^	5.01 × 10^−2^	1.42 × 10^−1^	1.42 × 10^−1^	1.68 × 10^−4^	7.17 × 10^−4^	5.00 × 10^−1^	1.92 × 10^−1^
Cr	7.92 × 10^1^	3.63 × 10^0^	1.95 × 10^−4^	2.72 × 10^−5^	7.53 × 10^−4^	7.53 × 10^−4^	3.11 × 10^−6^	8.30 × 10^−7^	6.49 × 10^−2^	9.08 × 10^−3^	7.53 × 10^−3^	7.53 × 10^−3^	3.05 × 10^−4^	1.30 × 10^−3^	7.27 × 10^−2^	1.79 × 10^−2^
Hg	4.92 × 10^−1^	3.79 × 10^−2^	1.02 × 10^−6^	1.43 × 10^−7^	5.86 × 10^−6^	5.86 × 10^−6^	1.64 × 10^−8^	4.36 × 10^−9^	6.40 × 10^−3^	8.96 × 10^−4^	1.95 × 10^−5^	1.95 × 10^−5^	7.52 × 10^−7^	3.21 × 10^−6^	6.42 × 10^−3^	9.19 × 10^−4^
Pb	1.05 × 10^3^	6.22 × 10^1^	1.07 × 10^−2^	1.50 × 10^−3^	4.24 × 10^−2^	4.24 × 10^−2^	1.72 × 10^−3^	4.57 × 10^−4^	-	-	-	-	-	-	-	-
Mn	5.67 × 10^2^	2.97 × 10^1^	5.75 × 10^−3^	8.05 × 10^−4^	2.28 × 10^−2^	2.28 × 10^−2^	9.20 × 10^−5^	2.45 × 10^−5^	4.11 × 10^−2^	5.75 × 10^−3^	4.55 × 10^−1^	4.55 × 10^−1^	4.83 × 10^−6^	2.06 × 10^−5^	4.96 × 10^−1^	4.61 × 10^−1^
Ni	7.68 × 10^1^	3.26 × 10^0^	3.24 × 10^−4^	4.53 × 10^−5^	1.29 × 10^−3^	1.29 × 10^−3^	5.18 × 10^−6^	1.38 × 10^−6^	2.94 × 10^−2^	4.12 × 10^−3^	1.29 × 10^−1^	1.29 × 10^−1^	8.64 × 10^−5^	3.68 × 10^−4^	1.59 × 10^−1^	1.34 × 10^−1^
Sb	7.46 × 10^1^	4.26 × 10^0^	7.33 × 10^−4^	1.03 × 10^−4^	2.89 × 10^−3^	2.89 × 10^−3^	1.17 × 10^−5^	3.12 × 10^−6^	1.83 × 10^0^	2.56 × 10^−1^	1.45 × 10^−2^	1.45 × 10^−2^	1.44 × 10^−3^	6.12 × 10^−3^	1.85 × 10^0^	2.77 × 10^−1^
Se	9.39 × 10^1^	5.33 × 10^0^	9.06 × 10^−4^	1.27 × 10^−4^	3.59 × 10^−3^	3.59 × 10^−3^	1.45 × 10^−5^	3.86 × 10^−6^	1.81 × 10^−1^	2.54 × 10^−2^	1.79 × 10^−4^	1.79 × 10^−4^	2.13 × 10^−5^	9.07 × 10^−5^	1.81 × 10^−1^	2.56 × 10^−2^
Tl	1.00 × 10^1^	5.83 × 10^−1^	1.03 × 10^−4^	1.44 × 10^−5^	4.05 × 10^−4^	4.05 × 10^−4^	1.65 × 10^−6^	4.40 × 10^−7^	1.031 × 10^1^	1.44 × 10^0^	-	-	1.21 × 10^−3^	5.17 × 10^−3^	1.03 × 10^1^	1.45 × 10^0^
Sum			3.20 × 10^−2^	4.48 × 10^−3^	1.25 × 10^−1^	1.25 × 10^−1^	2.09 × 10^−3^	5.55 × 10^−4^	1.640 × 10^1^	2.30 × 10^0^	9.70 × 10^−1^	9.70 × 10^−1^	4.51 × 10^−3^	1.92 × 10^−2^	1.738 × 10^1^	3.28 × 10^0^

CDI: Chemical daily intake through ingestion (mg/kg); EC: Exposure concentration through inhalation (µg/m^3^); DAD: Dermally absorbed dose (mg/kg); Bold: Value above the safe level.

**Table 4:** Carcinogenic risks from toxic elements in PM_2.5._

Toxic Elements	95%UCL		CDI (mg/kg-Day)		EC (μg/m^3^)		DAD (mg/kg)		Carcinogenic Risk (CR)							
	(ng/mg)	(ng/m^3^)	Child	Adult	Child	Adult	Child	Adult	Child-Ing	Adult-Ing	Child-Inh	Adult-Inh	Child-Der	Adult-Der	Child	Adult
Al	1.59 × 10^3^	7.80 × 10^1^	1.02 × 10^−3^	5.69 × 10^−4^	3.90 × 10^−3^	1.56 × 10^−2^	1.63 × 10^−5^	1.73 × 10^−5^	-	-	-	-				
As	1.07 × 10^2^	5.97 × 10^0^	9.17 × 10^−5^	5.13 × 10^−5^	3.63 × 10^−4^	1.45 × 10^−-3^	4.40 × 10^−6^	4.69 × 10^−6^	**1.37 × 10^−4^**	7.70 × 10^−5^	1.56 × 10^−6^	6.25 × 10^−6^	6.60 × 10^−6^	7.03 × 10^−6^	1.46 × 10^−4^	9.03 × 10^−5^
Be	2.78 × 10^−1^	1.37 × 10^−2^	1.85 × 10^−7^	1.04 × 10^−7^	7.32 × 10^−7^	2.93 × 10^−6^	2.96 × 10^−9^	3.16 × 10^−9^	-	-	1.76 × 10^−9^	7.03 × 10^−9^	-	-	1.76 × 10^−9^	7.03 × 10^−9^
Cd	6.31 × 10^1^	3.59 × 10^0^	3.07 × 10^−5^	1.72 × 10^−5^	1.21 × 10^−4^	4.85 × 10^−4^	4.91 × 10^−8^	5.23 × 10^−8^	-	-	2.18 × 10^−7^	8.73 × 10^−7^	-	-	2.18 × 10^−7^	8.73 × 10^−7^
Cr	7.92 × 10^1^	3.63 × 10^0^	1.67 × 10^−5^	9.34 × 10^−6^	6.45 × 10^−5^	2.58 × 10^−4^	2.67 × 10^−7^	2.84 × 10^−7^	8.34 × 10^−6^	4.67 × 10^−6^	5.42 × 10^−6^	2.17 × 10^−5^	5.34 × 10^−6^	5.69 × 10^−6^	1.91 × 10^−5^	3.20 × 10^−5^
Hg	4.92 × 10^−1^	3.79 × 10^−2^	8.78 × 10^−8^	4.91 × 10^−8^	5.02 × 10^−7^	2.01 × 10^−6^	1.40 × 10^−9^	1.50 × 10^−9^	-	-	-	-	-	-	-	-
Pb	1.05 × 10^3^	6.22 × 10^1^	9.19 × 10^−4^	5.14 × 10^−4^	3.64 × 10^−3^	1.45 × 10^−2^	1.47 × 10^−4^	1.57 × 10^−4^	**2.57 × 10^−4^**	**1.44 × 10^−4^**	2.91 × 10^−7^	1.16 × 10^−6^	4.12 × 10^−5^	4.39 × 10^−5^	2.99 × 10^−4^	1.89 × 10^−4^
Mn	5.67 × 10^2^	2.97 × 10^1^	4.93 × 10^−4^	2.76 × 10^−4^	1.95 × 10^−3^	7.80 × 10^−3^	7.88 × 10^−6^	8.40 × 10^−6^	-	-	-	-	-	-	-	-
Ni	7.68 × 10^1^	3.26 × 10^0^	2.77 × 10^−5^	1.55 × 10^−5^	1.11 × 10^−4^	4.43 × 10^−4^	4.44 × 10^−7^	4.73 × 10^−7^	-	-	2.66 × 10^−8^	1.06 × 10^−7^	-	-	2.66 × 10^−8^	1.06 × 10^−7^
Sb	7.46 × 10^1^	4.26 × 10^0^	6.28 × 10^−5^	3.52 × 10^−5^	2.48 × 10^−4^	9.92 × 10^−4^	1.00 × 10^−6^	1.07 × 10^−6^	-	-	-	-	-	-	-	-
Se	9.39 × 10^1^	5.33 × 10^0^	7.76 × 10^−5^	4.35 × 10^−5^	3.08 × 10^−4^	1.23 × 10^−3^	1.24 × 10^−6^	1.32 × 10^−6^	-	-	-	-	-	-	-	-
Tl	1.00 × 10^1^	5.83 × 10^−1^	8.84 × 10^−6^	4.95 × 10^−6^	3.47 × 10^−5^	1.39 × 10^−4^	1.41 × 10^−7^	1.51 × 10^−7^	-	-	-	-	-	-	-	-
Sum									**4.03 × 10^−4^**	**2.26 × 10^−4^**	7.52 × 10^−6^	3.01 × 10^−5^	5.31×10^−5^	5.66 × 10^−5^	4.64 × 10^−4^	3.12 × 10^−4^

Bold: Value above the safe level.
